# Strategic priorities and barriers to an effective anti-doping programme: views and opinions of European NADO leaders

**DOI:** 10.3389/fspor.2026.1812825

**Published:** 2026-06-02

**Authors:** Fredrik Lauritzen, Michael Petrou

**Affiliations:** 1 Science and Medicine, Anti-doping Norway, Oslo, Norway; 2 Cyprus Anti-Doping Authority, Nicosia, Cyprus

**Keywords:** anti-doping education, anti-doping organization, contamination, dietary supplement, doping control, WADA

## Abstract

National Anti-Doping Organizations (NADOs) play significant role in implementing the World Anti-Doping Code, yet little empirical research exists regarding their strategic priorities and perceptions about national and global anti-doping measures. This study explored the views of European NADO leaders on key areas for improving anti-doping programmes, perceived deterrent factors, and operational challenges. An online survey was distributed to 34 NADOs ahead of a 2025 European NADO conference; 32 (91%) NADO leaders responded. Across NADOs of all sizes, increased funding was the most frequently selected priority for improving both global (72%) and national (47%) anti-doping systems. Strengthening intelligence and investigation capacity (63%) and implementing values-based education (50%) were also considered essential for global improvement, while, improving testing effectiveness (41%) and expanding education programmes (31%) were key strategic aims within their own NADO. In contrast, increasing the number of tests or enhancing laboratory analyses were generally not considered a priority. More intensive anti-doping education was viewed as the most effective deterrent to prevent anti-doping rule violations (52%), followed by a higher perceived risk of detection (35%). Regarding risks of contamination, dietary supplements were widely regarded as high-risk for athletes in their country (68%), while environmental contamination was considered minimal risk. Major barriers to achieving strategic goals included insufficient funding (50%), limited human resources (31%), and inadequate political support (28%). These findings provide evidence-based insights into the shared challenges and strategic needs of European NADOs, informing future policy development and capacity building within anti-doping systems.

## Introduction

Athletes have a longstanding history of using performance-enhancing substances to boost their abilities and gain a competitive edge (1). The first systematic measures aiming at combating doping started in the 1960s when the International Olympic Commission established a medical commission mandated to develop a list of substances and methods prohibited to use by athletes. The Council of Europe's Anti-Doping Convention of 1989 marked the first step on the site of the states toward international harmonisation against doping in sport (2). The Convention provides for obligations for the governments and requires that governmental departments or agencies, like the departments responsible for public health, medical care, customs, police, sport, education, and the national federations all need to work together constructively to achieve best results, making it harder to obtain and use banned substances. Even though the Convention does not propose a single operative method, parties should ensure the practical implementation of the Convention and, in that respect, establish a national responsible body, to ensure consistency across all sports at the national level.

Following the establishment of the World Anti-Doping Agency (WADA) in 1999 and the entry into force of the first World Anti-Doping Code (WADC) in 2004, each country is required to designate a National Anti-Doping Organization (NADO) defined as an entity “(…) possessing the primary authority and responsibility to adopt and implement anti-doping rules, direct the collection of samples, the management of test results, and the conduct of hearings at the national level” (3). NADOs ensure that anti-doping work is planned and executed independent from sport organisations, which may be more interested in protecting their own interest than to detect and deter doping practices (4). As such, NADOs play a key role both at national and international levels in combating doping in line with the WADC and International Standards. NADOs conduct testing of athletes, conduct investigations, handle results management, and act as the central body for promoting education and prevention strategies. Moreover, through groups like the Monitoring Group to the Council of Europe's Anti-Doping Convention or WADA's NADO Expert Advisory Group, NADOs have the potential to influence international policies for the fight against doping in sport.

Regardless of the significant role of the NADOs and the experience and expertise of NADO staff, there is a lack of research studies examining their views, experiences and professional challenges related to the fight against doping in sport.

The aim of this study is to examine the views and opinions of European NADO leaders on the priorities and barriers to effective anti-doping programmes, in their own countries but also globally.

## Method

The study is based on data collected through an online survey among executive representatives (i.e., NADO leaders) of European NADOs.

### Participants

An online survey was distributed to the head of delegation of each registered NADO one week preceding the conference “Bringing European National Anti-Doping Organisations Together to Strengthen National Systems” which was jointly organised by the Council of Europe and Anti-Doping Norway in Bergen, Norway in June 2025. The recipients of the survey were in most cases the CEO or President of the NADO. In total, 34 NADOs that have registered for the conference were invited to participate in the study, of which 32 (91%) NADO leaders completed most or all survey questions.

### Survey instrument

A short questionnaire specifically for the purposes of the study was developed. The question regarding effective deterrent factors was inspired by a similar question from the 2022 U.S. Anti-doping Agency Athlete Perception Study (5) and adjusted to fit the context of this study.

The survey included mostly closed-response, multiple-choice questions to gather quantitative data, allowing for easy comparison and facilitating a high response rate and a relatively large sample size. The survey consisted of nine questions with an emphasis on strategic priorities and barriers to an effective anti-doping programme. Questions and response options were developed by the authors and piloted among senior level NADO staff not involved in the study to ensure that the questions were easily understood and that the most relevant alternatives were available in the multiple-choice format.

All responses were collected anonymously. To facilitate comparison of NADOs with varying sizes and resource levels, organizations were classified according to staff size using the following categories: “Small” (1–10 employees), “Medium” (11–20 employees), and “Large” (more than 20 employees).

The following questions were included in the survey: 1) “What is the size of your anti-doping organisation in terms of staff/personnel (excluding DCOs)”, 2) “In your opinion, what are the five most important areas for improving the global anti-doping programme?”, 3) “In the previous question you selected the following five most important areas for improving the global anti-doping programme. Please rank them in a prioritised order, where 1 is the most important area, by moving each alternative up or down”, 4) “What are the three main strategic priorities to improve the anti-doping programme in your NADO?”, 5) “In the previous question you selected the following three most important areas for improving the anti-doping programme in your NADO. Please rank them in a prioritised order where 1 is the most important area, by moving each alternative up or down”, 6) “In your opinion, which of the following do you think is most effective in deterring athletes from intentionally violating the anti-doping rules in the future?”, 7) “To what extent do each of the following possible sources of contamination of Prohibited substances pose a risk to athletes in your country”, 8) “Which are the most significant barriers to you achieving your strategic priorities for improving your anti-doping programme? Please select minimum one and maximum three of the following items,” and 9) “Which operational area in your NADO would be the most important to strengthen for your organisation to achieve its strategic priorities? Please select one alternative”.

For questions 1, 2, 4, 6, 8 and 9, predefined response options were given. For question 3 and 5, the respondents were asked to rank in a prioritized order the alternatives previously selected in question 2 and 4, respectively. For question 7, a four-point Likert scale (not at all; some risk; moderate risk; high risk) was employed to assess respondents' perceptions regarding the risk of contamination doping from sources such as meat, supplements, pharmaceuticals, intimate contact, and environmental factors. For questions 8 and 9, in addition to the predefined response options, respondents were given the opportunity to include other alternatives through an “Other, please specify” option. At the end of the survey, the participants were given the option to add a comment.

The full survey with all questions and response options are available as supplementary material (Supplementary File S1).

### Data analysis

Categorical variables are presented as counts and percentages. IBM SPSS Statistics version 31 and Microsoft Excel were used for processing and analysis of data.

### Research ethics

Research approval to conduct the study was granted by the Cyprus National Bioethics Committee (EEBK 2025.01.219).

## Results

In total, 32 (91%) out of 34 conference participants invited to participate in the study completed all or most of the survey questions. Of these 28% (*n* = 9) represented “small-size” NADOs, that is NADOs with a staff count between 1 and 10 people, 44% (*n* = 14) represented “medium-size” NADOs that is NADOs of 11–20 people, and 28% (*n* = 9) represented “large-size” NADOs with more than 20 people in their staff.

### Areas to improve the global anti-doping programme

When the participants were asked to select from a list of 15 areas, the five areas selected by most NADO representatives for improving the global anti-doping programme, was increase funding to anti-doping (72%, *n* = 23), strengthen the intelligence and investigation capacity of Anti-Doping Organisations (ADOs) (63%,*n* = 20), provide values-based anti-doping education (50%, *n* = 16), operational independence (50%, *n* = 16) and global coordination and harmonization of anti-doping regulations across countries and sports (47%, *n* = 15) (Table 1). Overall, the least important areas for improving the global anti-doping programme were to increase the overall testing numbers and improving laboratory sample analyses (both selected by only two respondents as among the top five most important areas).

**Table 1:** Prioritized areas to improve the global anti-doping programme.

Prioritized areas	Total (*n* = 32)	Small (*n* = 9)	Medium (*n* = 14)	Large (*n* = 9)
Increase funding to anti-doping	**72%**	78%	64%	78%
Strengthen the intelligence and investigation capacity of ADOs	**63%**	56%	71%	56%
Operational independence of NADOs	**50%**	67%	50%	25%
Provide values-based anti-doping education and awareness raising programmes for athletes and support personnel	**50%**	44%	57%	44%
Global coordination and harmonization of anti-doping regulations across different countries and sports	**47%**	56%	29%	67%
Increase collaboration between NADOs at the global level	**41%**	44%	29%	44%
Increasing the effectiveness of testing	**34%**	11%	36%	56%
Increase cooperation with International Federations	**28%**	33%	36%	11%
Geopolitical issues	**25%**	44%	21%	11%
Reduce the number of unintentional doping cases	**22%**	0%	29%	33%
Capacity building of smaller/less resourced ADOs	**19%**	22%	21%	11%
A more athlete-centred anti-doping programme	**19%**	11%	29%	11%
Increase cooperation with WADA	**19%**	11%	14%	33%
Increase the overall testing numbers	**6%**	11%	7%	0%
Improving laboratory sample analysis, e.g., for novel substances and methods	**6%**	11%	7%	0%

The proportion of respondents selecting each alternative as one of five most important areas to improve the global anti-doping programme. Data shown in bold for all organisations combined (total), and by the size of the NADO (small; medium; large). ADO, Anti-Doping Organisation; NADO, National Anti-Doping Organisation.

The NADO leaders had somewhat different opinions depending on the size of the organisation they represented (Table 1), however there were generally consensus regarding the most and least selected areas. Increase funding to anti-doping was the most selected area by both “small” (78%) and “large” (78%) NADOs, while among “medium-sized” NADOs funding came second (64%) to strengthening the intelligence and investigation capacity of ADOs (71%). At the opposite end, increase in overall testing numbers and improving laboratory analysis was considered as the least important areas for improving the global anti-doping programme independent of the NADO size.

When asked to rank the five most important areas previously selected by importance (answered by 28 respondents), operational independence was ranked first by seven NADOs, followed by increased funding to anti-doping (*n* = 5), and provide values-based anti-doping education (*n* = 3).

### Main strategic priorities to improve the national anti-doping programme

Overall, the three strategic priorities to improve the national anti-doping programme selected by most NADO representatives were increase funding to anti-doping (47%, *n* = 15 of 32), increasing the effectiveness of testing, i.e., increasing the detection ratio of collected samples (41%, *n* = 13 of 32) and provide values-based anti-doping education and awareness raising programmes for athletes and support personnel (31%, *n* = 10 of 32) (Table 2). The three least important strategic priorities of the provided alternatives were increase bi-lateral and/or multilateral collaboration between my NADO and other NADOs (9%, *n* = 3 of 32), more effective use of current financial resources (6%, *n* = 2 of 32) and increase the overall testing numbers (3%, *n* = 1 of 32).

**Table 2:** Prioritized areas to improve the national anti-doping programme.

Prioritized areas	Total (*n* = 32)	Small (*n* = 9)	Medium (*n* = 14)	Large (*n* = 9)
Increase funding to anti-doping	**47%**	56%	36%	56%
Increasing the effectiveness of testing, i.e., increasing the detection ratio of collected samples	**41%**	33%	36%	56%
Provide values-based anti-doping education and awareness raising programmes for athletes and support personnel	**31%**	44%	21%	33%
National legislation	**28%**	56%	21%	11%
Preparing for the updated WADC	**28%**	11%	29%	44%
Operational independence	**19%**	11%	29%	11%
Internal capacity building	**19%**	11%	29%	11%
Increase anti-doping research activity within the NADO	**19%**	11%	14%	33%
Organisation of the NADO (i.e., organizational structure and staff competency)	**16%**	33%	7%	11%
Strengthen the Intelligence and investigation capacity of the NADO	**13%**	0%	21%	11%
Reduce the number of unintentional doping cases	**13%**	11%	14%	11%
Increase bi-lateral/multi-lateral collaboration between my NADO and other NADOs	**9%**	11%	7%	11%
More effective use of current financial resources	**6%**	0%	14%	0%
Increase the overall testing numbers	**3%**	11%	0%	0%

The proportion of respondents selected each alternative as one of three most important areas to improve the anti-doping programme in their own NADO. Data shown in bold for all organisations combined (total), and by the size of the NADO (small; medium; large). AAF, adverse analytical finding; NADO, National Anti-Doping Organisation.

Increase funding to anti-doping was the most selected strategic priority independent of NADO size, while the importance of other strategic priorities varied between the NADO categories (Table 2).

### Effective deterrent factors

More intensive anti-doping education (e.g., annual online education, informing about adverse effects of Prohibited substances, values-based education, education from peers etc.) was considered the most effective factor in deterring athletes from intentionally violating the anti-doping rules in the future (52%, *n* = 16 of 31).

A high risk of detection following a more comprehensive/effective testing programme (e.g., unpredictable testing, more frequent testing, athlete biological profiles, long-term storage and re-analysis of samples) was considered the most effective deterrent among 35% of the respondents (*n* = 11 of 31), while stricter punishment/sanctions following rule violations (e.g., public disclosure of positive test results, loss of medals/sponsors/prize money, ban from sport, media scrutiny etc.) was considered the most effective deterrent by 13% (*n* = 4 of 31). One survey respondent did not answer the deterrence question.

### Sources of contamination

**Figure 1:**
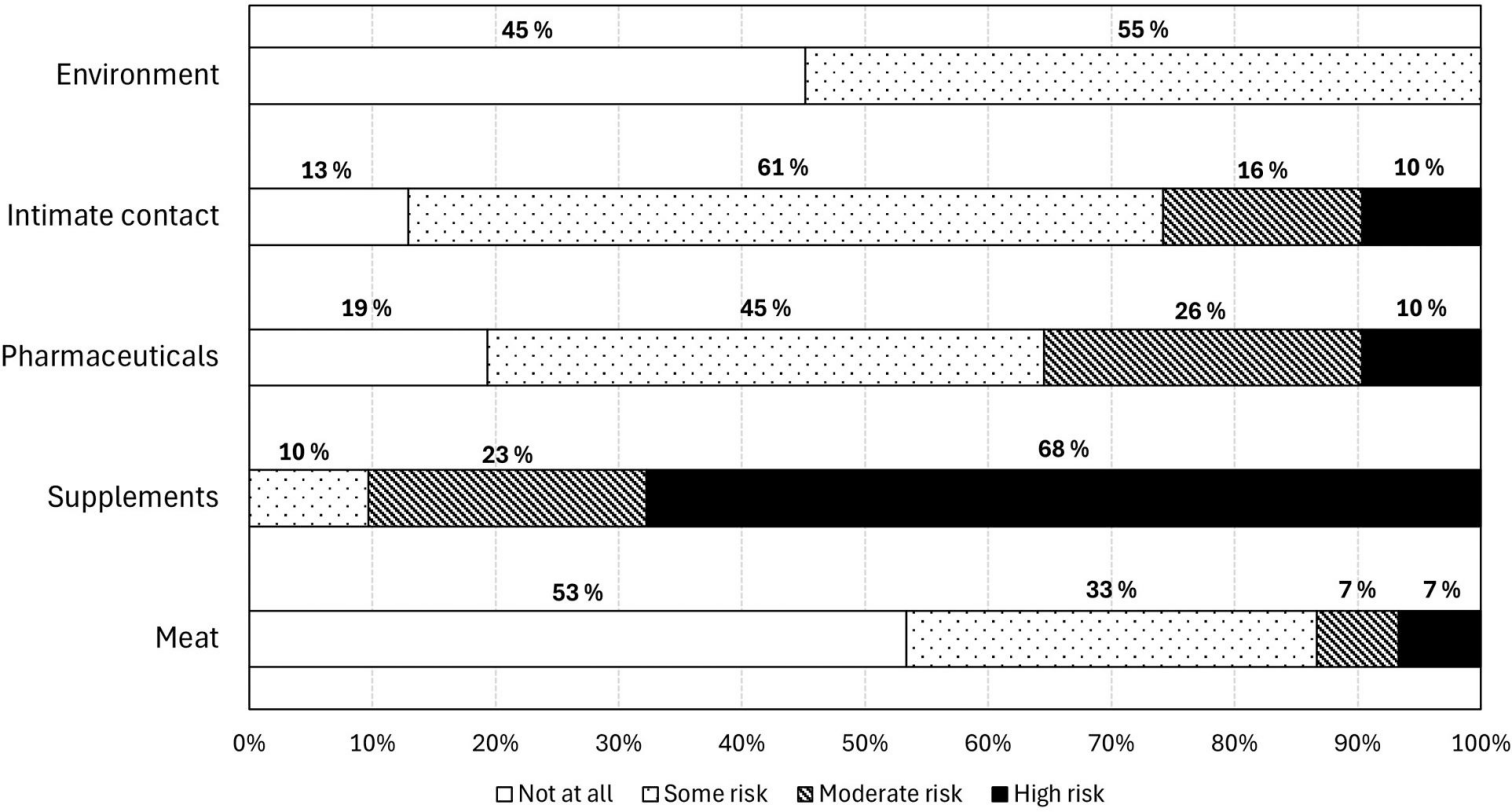
To what extend each of the following possible sources of contamination of prohibited substances pose a risk to athletes in your country? environmental contamination may for example include water or soil contamination. Based on responses from 31 participants.

Of the various potential sources of contamination, dietary supplements were regarded as the most significant, where 68% of respondents identified contaminated supplements as a considerable risk to athletes in their country, while 23% assessed the risk as moderate (Figure 1). No NADO leaders believed that supplements did not constitute a risk at all.

Other sources of contamination also raised notable concerns (Figure 1). Pharmaceuticals were seen as a high or moderate risk to athletes in their country by 36% of the respondents, followed by 26% for intimate contact and 14% for meat. In contrast, contamination of prohibited substances from the environment was not perceived as a significant risk.

### Barriers and operational areas to strengthen

Inadequate funding (50%, *n* = 16), a general lack of human resources and/or insufficient staff competency (31%, *n* = 10), a lack of political will or support (28%, *n* = 9) and inadequate national legislation (19%, *n* = 6) were considered the most significant barriers to the NADO representatives' achieving their strategic priorities for improving their anti-doping programmes. The remaining response options were selected by less than four participants.

Overall, the most selected operational areas to strengthen for the NADO to achieve its strategic priorities were education and prevention (25%, *n* = 7), intelligence and investigations (18%, *n* = 5) and legal and regulatory affairs, including policy development and compliance (18%, *n* = 5). The remaining response options were selected by three or fewer participants. Four NADO representatives did not respond to the question.

## Discussion

This study provides a descriptive snapshot of European NADO leaders views and perceptions on important and timely anti-doping issues. It highlights areas considered important to strengthen global and national anti-doping work, factors perceived by European NADO leaders to be effective in deterring doping, and the issue of contamination, which recently has received much attention in the anti-doping community (6).

According to the survey findings, increased funding was consistently, irrespective of NADO size, identified among the provided response alternatives as the most important to strengthening both global and national programmes. This finding is in accordance with previous research reporting a lack of financial resources as a major barrier to employ effective education programmes (7), and that variations in organisational and human resources among anti-doping organisations may cause capacity constraints and a lack of harmonization (8). It is well known that financial resources available to NADOs vary, also within Europe. In general, larger countries have larger NADOs with higher annual budgets than smaller countries. According to data collected by the Council of Europe, Germany and France, which both qualifies as “large” NADOs using the definition applied in this study (i.e., >20 staff), reported annual budgets in 2023 of approximately EUR 12,500,000 (9). In comparison, “medium” sized NADOs (11–20 staff) Austria and Denmark reported budgets of approximately EUR 4,000,000, whereas “small” sized NADOs (1–10 staff) Cyprus and Estonia had annual budgets below EUR 1,000,000. Limited financial resources could make it challenging to run an effective anti-doping programme in line with all mandatory requirements and recommendations of the World Anti-Doping Programme (WADP), which has become increasingly complex in recent years requiring knowledge and competencies in multiple disciplines (3, 10). Also, as the requirements set by WADA apply to all NADOs irrespective of their size, such as within the area of testing and sample analysis (11), smaller organisations and those based in low-income countries may face challenges balancing their activities with limited financial and human resources. An example of this challenge was evident in a recent study examining the results management process, another key area for NADOs. The study found significant differences in delays in issuing the first-instance hearing decision and notifying the athlete in the course of anti-doping rule violations results management based on the United Nations WESP 2022 income-based country classification (12). Compared to high-income countries, significantly more countries from lower-middle and upper-middle reported such delays.

A call for more resources to develop and manage anti-doping programmes is not new. A 2013 report to the WADA Executive Committee from a working group tasked with investigating the shortcomings of testing programmes noted a lack of commitment towards achieving doping-free sport. It highlighted an “…unwillingness to commit the necessary informed intelligence, effective actions, and other resources to the fight against doping in sport” (pp. 3, 10), and that “Governments should ensure that their NADOs are independent and adequately funded…” [p. 7, (13)]. Traditionally, increased funding has been used for more testing; however, critics argue that larger NADO budgets in many countries have not led to improved detection rates (14). This is supported by recent global test statistics which show that rising testing numbers have not led to more adverse findings or anti-doping violations (15), and furthermore by a study from a NADO examining seventeen years of national testing data showing no correlation between sample collection and detection rates, indicating that increased funding is only effective if used strategically (16). It should, however, be remembered that anti-doping programmes are not only aimed at detecting adverse analytical findings through doping controls (15), but also to serve as both deterrents and preventative measures (17–19), with their effectiveness being closely linked to the level of resources available for implementation.

According to the findings, European NADO leaders were in general not having the need for more testing in mind when requesting increased funding, as Increasing overall testing numbers were not considered as a priority for enhancing global or national anti-doping programmes, with only one respondent (3%) selecting this among the most important factors for improving the national anti-doping programme. This could be possibly explained by the fact that testing, on average is already seizing about half of NADO budgets (20). Improving the effectiveness of testing, however, was deemed important by about one third of respondents. Key to improving testing effectiveness is strengthening of intelligence and investigation capabilities (16), an area considered the second most important factor to improve global anti-doping programmes. In recent years, the application of forensic techniques in doping control, as well as the incorporation of intelligence into risk assessment and test planning to enhance testing efficiency, has received increasing attention (16, 21). Intelligence and investigations are now key parts of the WADP and expected to be strengthened further with the new International Standard for Intelligence and Investigations that comes into effect from 2027 (22). When considering their own NADO, however, only 13% of the respondents considered strengthening intelligence and investigation capacities a priority. This discrepancy warrants further research. A possible explanation could be that European NADO leaders believe that they are already using Intelligence and investigations sufficiently, possibly due to recent extensive capacity building in the region (23).

One of the most important topics in relation to anti-doping that is discussed since the establishment of WADA more than 25 years ago, is the NADO operational independence and the avoidance of real or perceived conflicts of interest. It is acknowledged that undue influence has negatively impacted anti-doping efforts. The Institute of National Anti-Doping Organisations (24) has argued that “policies resembling a fox guarding the henhouse where sport polices itself, along with threats from lack of independence, undue state influence, and fraud, are part of the anti-doping history which has generated change” and have pointed out the importance of good governance and independence in addressing potential conflict of interest where doping control activities are compromised by interests other than detecting doping, like “the glory of a government, the commercial interests of sport, such as an International Federation, or the reputation of a sport/ country above the protection of athletes and fair and honest sport”. Interestingly, 50% of the respondents to our questionnaire agreed that NADO operational independence is one of the most important areas for the improvement of the global anti-doping programme.

When comparing whether improving education, detection or sanctioning was the most effective in preventing athletes from intentionally violating anti-doping rules in the future, more intensive anti-doping education was considered the most effective factor, selected by 52% of the European NADO leaders. Providing values-based anti-doping education and awareness raising programmes for athletes and support personnel was also ranked high both with respect to improving global and national anti-doping efforts. Indeed, providing information to increase knowledge and raise awareness as well as the provision of education that focuses on values to change behaviour, for athletes and their support persons is a key element of anti-doping programmes and compliments the detection and deterrence effect of testing (25). Education was also considered as one of the five most important areas for improving anti-doping programmes. Effectiveness of many prevention initiatives, however, is not well known and future studies should aim to evaluate how these affect anti-doping knowledge, attitudes and clean sport behaviours (26).

The present study is not free of limitations. The quantitative method facilitates straightforward comparison of respondents and accommodates larger sample sizes; however, predefined responses may introduce response bias if the respondents' perceptions are not fully represented in the provided options. Future studies using a mixed-methods or qualitative approach would be useful to determine if NADO leaders highlight priorities not identified in this study. Moreover, the findings are derived from a limited number of participants, of which all represent European NADOs. Previous research has indicated significant variations in clean sport attitudes and perceived legitimacy of anti-doping practices among athletes in different countries and regions (27, 28). While all NADOs follow the World Anti-Doping Programme, regional differences in culture, structure, and economy may shape their views on priorities, challenges, and programme effectiveness. Therefore, including only organisations from one continent, may result in findings that are not widely generalizable. To develop a more comprehensive understanding of strategic priorities and barriers among NADOs globally, similar research should be conducted in other regions, such as in Asia, Africa and the Americas.

It should also be noted that the study took place during a transitional phase for the global anti-doping movement, with data collected during a period with ongoing preparations for an updated WADC and International Standards. These new regulations, mandatory requirements, and guidelines, scheduled to take effect on January 1st, 2027, are anticipated to influence the priorities of all affected stakeholders, including NADOs. Reevaluating strategic priorities among European NADO leaders in the future could help determine how a new set of regulations influence their views of both the international and national anti-doping programmes.

To the best of our knowledge, this is the first study to examine the views of European NADO leaders on how to improve anti-doping programmes in the countries and globally and what are the barriers. The findings of the survey can assist policy making organisations like the Council of Europe, WADA and the NADOs themselves to further improve the anti-doping programmes and develop and/or implement effective anti-doping programmes.

## Data Availability

The raw data supporting the conclusions of this article will be made available by the authors, without undue reservation.
