# Supplementary material for: Strategic priorities and barriers to an effective anti-doping programme: views and opinions of European NADO leaders
Source: Front Sports Act Living. 2026 Jun 2;8:1812825. doi: 10.3389/fspor.2026.1812825 (PMC13269393; doi:10.3389/fspor.2026.1812825)
Supplement: Supplementary file 1 [file Supplementaryfile1.docx]

**Survey on strategic priorities and barriers for effective national and global anti-doping programmes**

1. What is the size of your national anti-doping organisation in terms of staff/personnel (excluding DCOs)

- *1-10*
- *11-20*
- *More than 20*

1. In your opinion, what are the **five most important areas** for improving the **global** anti-doping program?
   - *Operational independence of NADOs*

- *Global coordination and harmonization of anti-doping regulations across different countries and sports*
- *Capacity building of smaller/less resourced ADOs*
- *Increase funding to anti-doping*
- *Increase the overall testing numbers*
- *Increasing the effectiveness of testing, i.e., increasing the detection ratio of collected samples*
- *Improving laboratory sample analysis, e.g., for novel substances and methods*
- *Strengthen the Intelligence and investigation capacity of ADOs*
- *Reduce the number of unintentional doping cases, e.g., AAF caused by contamination from supplements, medicines, food and intimate contact*
- *Increase bi-lateral and/or multi-lateral collaboration between NADOs at the global level, including sharing of experiences and good practice*
- *Provide values-based anti-doping education and awareness raising programs for athletes and support personnel*
- *A more athlete-centred anti-doping program*
- *Increase cooperation with WADA*
- *Increase cooperation with International Federations*
- *Geopolitical issues*

1. In the previous question, you selected the following five most important areas for improving the **global** anti-doping program. *Please rank them in a prioritized order where 1 is the most important area by moving each alternative up or down*
   - *National legislation*
   - *Operational independence of NADOs*

- *Global coordination and harmonization of anti-doping regulations across different countries and sports*
- *Capacity building of smaller/less resourced ADOs*
- *Increase funding to anti-doping*
- *Increase the overall testing numbers*
- *Increasing the effectiveness of testing, i.e., increasing the detection ratio of collected samples*
- *Improving laboratory sample analysis, e.g., for novel substances and methods*
- *Strengthen the Intelligence and investigation capacity of ADOs*
- *Reduce the number of unintentional doping cases, e.g., AAF caused by contamination from supplements, medicines, food and intimate contact*
- *Increase bi-lateral and/or multi-lateral collaboration between NADOs at the global level, including sharing of experiences and good practice*
- *Provide values-based anti-doping education and awareness raising programs for athletes and support personnel*
- *A more athlete-centred anti-doping program*
- *Increase cooperation with WADA*
- *Increase cooperation with International Federations*
- *Geopolitical issues*

1. *What are the* ***three main strategic priorities*** *to improving the anti-doping program in* ***your NADO****?*

- *National legislation*
- *Operational independence*
- *Increase funding to anti-doping*
- *More effective use of current financial resources*
- *Preparing for the updated WADC*
- *Organisation of the NADO (i.e., organizational structure and staff competency)*
- *Internal capacity building*
- *Increase the overall testing numbers*
- *Increasing the effectiveness of testing, i.e., increasing the detection ratio of collected samples*
- *Strengthen the Intelligence and investigation capacity of the NADO*
- *Reduce the number of unintentional doping cases, e.g., AAF caused by contamination from supplements, medicines, food and intimate contact*
- *Increase bi-lateral and/or multi-lateral collaboration between my NADO and other NADOs*
- *Provide values-based anti-doping education and awareness raising programs for athletes and support personnel*
- *Increase anti-doping research activity within the NADO*

1. *In the previous question you selected the following* ***three most important*** *areas for improving the anti-doping program in* ***your NADO****. please rank them in a prioritized order where 1 is the most important area, by moving each alternative up or down*
   - *National legislation*
   - *Operational independence*
   - *Increase funding to anti-doping*
   - *More effective use of current financial resources*
   - *Preparing for the updated WADC*
   - *Organisation of the NADO (i.e., organizational structure and staff competency)*
   - *Internal capacity building*
   - *Increase the overall testing numbers*
   - *Increasing the effectiveness of testing, i.e., increasing the detection ratio of collected samples*
   - *Strengthen the Intelligence and investigation capacity of the NADO*
   - *Reduce the number of unintentional doping cases, e.g., AAF caused by contamination from supplements, medicines, food and intimate contact*
   - *Increase bi-lateral and/or multi-lateral collaboration between my NADO and other NADOs*
   - *Provide values-based anti-doping education and awareness raising programs for athletes and support personnel*
   - *Increase anti-doping research activity within the NADO*
2. In your opinion, which of the following do you think is **most effective** in deterring athletes from intentionally violating the anti-doping rules in the future?

- More intensive anti-doping education (e.g., annual online education, informing about adverse effects of Prohibited substances, values-based education, education from peers etc.)
- A high risk of detection following a more effective testing programme (e.g., unpredictable testing, more frequent testing, ABP, long term storage and re-analysis of samples, etc.)
- Stricter punishment/sanctions following rule violations (e.g., public disclosure of positive test results, loss of medals/sponsors/prize money, ban from sport, media scrutiny etc).

1. To what extent do each of the following possible sources of contamination of Prohibited substances pose risk to athletes in your country?

|  | Not at All | Some risk | Moderate risk | High risk |
| --- | --- | --- | --- | --- |
| Risk of contamination from meat |  |  |  |  |
| Risk of contamination from supplements |  |  |  |  |
| Risk of contamination from pharmaceuticals |  |  |  |  |
| Risk of contamination from intimate contact |  |  |  |  |
| Risk of contamination from the environment (e.g., water or soil contamination) |  |  |  |  |

1. Which are the **most significant barriers** to you achieving **your strategic priorities** for improving your anti-doping programme? *Please select minimum 1 and maximum 3 of the following alternatives.*

- *Inadequate national legislation*
- *Lack of political will or support*
- *Inadequate funding*
- *Limitations in testing technology*
- *Human right issues*
- *Inconsistent international cooperation*
- *Normalization of doping (e.g., cultural acceptance of doping)*
- *General lack of human resources, insufficient staff competency*
- *Geopolitical issues*
- *Lack of cooperation with national sport bodies and/or sport federations*
- *Other (please specify)*

1. *Which operational area in your NADO would be the most important to strengthen for your organisation to achieve its strategic priorities? Please select one alternative.*

- Test operations and planning
- Education and prevention
- Intelligence and Investigations
- Medicine, incl. TUE management
- Innovation and research
- Communications
- International and Diplomatic affairs
- Results Management
- Legal and regulatory affairs, including policy development and compliance
- Administrative functions (e.g., office management, IT, HR - human resources, finance etc).
- Other, please specify

1. Is there anything else you would like to add?

(optional free text)
